# Development and validation of an artificial intelligence system for grading colposcopic impressions and guiding biopsies

**DOI:** 10.1186/s12916-020-01860-y

**Published:** 2020-12-22

**Authors:** Peng Xue, Chao Tang, Qing Li, Yuexiang Li, Yu Shen, Yuqian Zhao, Jiawei Chen, Jianrong Wu, Longyu Li, Wei Wang, Yucong Li, Xiaoli Cui, Shaokai Zhang, Wenhua Zhang, Xun Zhang, Kai Ma, Yefeng Zheng, Tianyi Qian, Man Tat Alexander Ng, Zhihua Liu, Youlin Qiao, Yu Jiang, Fanghui Zhao

**Affiliations:** 1 Department of Epidemiology and Biostatistics, School of Population Medicine and Public Health, Chinese Academy of Medical Sciences and Peking Union Medical College, Beijing, 100730 China; 2 Department of Cancer Epidemiology, National Cancer Center/National Clinical Research Center for Cancer/Cancer Hospital, Chinese Academy of Medical Sciences and Peking Union Medical College, Beijing, 100021 China; 3 School of Public Health, Dalian Medical University, Dalian, China; 4 Diagnosis and Treatment for Cervical Lesions Center, Shenzhen Maternity & Child Healthcare Hospital, Shenzhen, China; 5 Tencent Jarvis Lab, Shenzhen, China; 6 Zonsun Healthcare, Shenzhen, China; 7 Center for Cancer Prevention Research, Sichuan Cancer Hospital & Institute, Sichuan Cancer Center, School of Medicine, University of Electronic Science and Technology of China, Chengdu, China; 8 Tencent Healthcare, Shenzhen, China; 9 Jiangxi Maternal and Child Health Hospital, Nanchang, China; 10 Chengdu Women’s and Children’s Central Hospital, School of Medicine, University of Electronic Science and Technology of China, Chengdu, China; 11 Chongqing University Cancer Hospital, Chongqing, China; 12 Cancer Hospital of China Medical University, Liaoning Cancer Hospital & Institute, Shenyang, China; 13 Affiliated Cancer Hospital of Zhengzhou University/Henan Cancer Hospital, Zhengzhou, China; 14 Department of Pathology, National Cancer Center/National Clinical Research Center for Cancer/Cancer Hospital, Chinese Academy of Medical Sciences and Peking Union Medical College, Beijing, China; 15 Department of Gynecology, Shenzhen Maternity & Child Healthcare Hospital, Shenzhen, China

**Keywords:** Artificial intelligence, Cervical cancer prevention, Colposcopy diagnosis and biopsy, Global elimination of cervical cancer

## Abstract

**Background:**

Colposcopy diagnosis and directed biopsy are the key components in cervical cancer screening programs. However, their performance is limited by the requirement for experienced colposcopists. This study aimed to develop and validate a Colposcopic Artificial Intelligence Auxiliary Diagnostic System (CAIADS) for grading colposcopic impressions and guiding biopsies.

**Methods:**

Anonymized digital records of 19,435 patients were obtained from six hospitals across China. These records included colposcopic images, clinical information, and pathological results (gold standard). The data were randomly assigned (7:1:2) to a training and a tuning set for developing CAIADS and to a validation set for evaluating performance.

**Results:**

The agreement between CAIADS-graded colposcopic impressions and pathology findings was higher than that of colposcopies interpreted by colposcopists (82.2% versus 65.9%, kappa 0.750 versus 0.516, *p* < 0.001). For detecting pathological high-grade squamous intraepithelial lesion or worse (HSIL+), CAIADS showed higher sensitivity than the use of colposcopies interpreted by colposcopists at either biopsy threshold (low-grade or worse 90.5%, 95% CI 88.9–91.4% versus 83.5%, 81.5–85.3%; high-grade or worse 71.9%, 69.5–74.2% versus 60.4%, 57.9–62.9%; all *p* < 0.001), whereas the specificities were similar (low-grade or worse 51.8%, 49.8–53.8% versus 52.0%, 50.0–54.1%; high-grade or worse 93.9%, 92.9–94.9% versus 94.9%, 93.9–95.7%; all *p* > 0.05). The CAIADS also demonstrated a superior ability in predicting biopsy sites, with a median mean-intersection-over-union (mIoU) of 0.758.

**Conclusions:**

The CAIADS has potential in assisting beginners and for improving the diagnostic quality of colposcopy and biopsy in the detection of cervical precancer/cancer.

**Supplementary Information:**

The online version contains supplementary material available at 10.1186/s12916-020-01860-y.

## Background

Cervical cancer results in high rates of morbidity and mortality worldwide and with a disproportionate effect on low- and middle-income countries (LMICs). Annually, over 85% of new cases and 87% of deaths occur in LMICs [1]. In 2018, the World Health Organization (WHO) called for action towards the reduction of cervical cancer cases based on proven strategies [2, 3]. One of these strategies is that 70% of women between the ages of 35 and 45 years receive screening. By 2030, 90% of women in this age group must be managed to achieve the goal of fewer than four new cases per 100,000 [4]. A major concern is the diagnostic ability to appropriately identify and manage women with abnormal screening status at the time of colposcopy. Accurate visual detection of underlying colposcopic abnormalities is critical in guiding biopsies for pathological confirmation. This detection is essential for the identification of high-grade squamous intraepithelial lesion or worse (HSIL+), lesions that require immediate treatment. However, current colposcopic evaluation presents challenges, especially in LMICs. These challenges include poor agreement (under 50%) between colposcopic impressions and pathological findings, a strong dependence on the subjective experience of operators, substantial variabilities among inter- and intra-operators, a large number of women with risk factors that warrant referral to colposcopy, and a shortage of experienced colposcopists [5–7].

In light of these challenges, the American Society for Colposcopy & Cervical Pathology (ASCCP) reviewed colposcopy standards to improve diagnostic performance by establishing a comprehensive evaluation based on available test results (cytology, human papillomavirus [HPV] status, and colposcopy impressions) [8, 9]. However, despite its widespread use, over the last decade, there has been little improvement in colposcopic performance, especially in LMICs [10].

Recently, artificial intelligence (AI) methods have shown potential in subjective imaging diagnoses for malignancies such as breast cancer, colorectal cancer, and gastrointestinal cancer [11–13]. The application of similar methods to colposcopic imaging is not yet widespread [14, 15]. In this study, we developed an AI method (Colposcopic Artificial Intelligence Auxiliary Diagnostic System [CAIADS]) for grading colposcopic impressions and guiding biopsies. We evaluated its performance on an independent validation set and compared it to colposcopy interpretations made by colposcopists.

## Methods

### The primary goals of the use of CAIADS

One of the primary goals in the application of CAIADS was to grade colposcopic impressions in accordance with the latest ASCCP colposcopy terminology: normal/benign, low-grade, high-grade, and cancer [16, 17]. The CAIADS was also expected to dichotomously grade colposcopic impressions into two hypothetical biopsy thresholds (low-grade or worse versus normal/benign, and high-grade or worse versus a less severe impression). These categories were used to find an appropriate colposcopically guided biopsy threshold and guide biopsies for detecting the clinically relevant endpoint (pathology-confirmed HSIL+).

### Study patients and design

Between January 12, 2018, and December 30, 2018, anonymized digital records of patients, including colposcopic images, non-image information (cytology and HPV status), and pathological results were retrospectively obtained from archived databases of six multicenter hospitals across China (Additional file 1, Table S1), including Shenzhen Maternity and Child Healthcare Hospital (SZMCHH). The pathological results were the gold standard for developing CAIADS and validate its diagnostic performance. The study was approved by the institutional review board (IRB) of SZMCHH. The need for informed consent was waived by the IRB of SZMCHH due to the retrospective nature of archived datasets and fully anonymized personal information.

All patients aged 24–65 years with indications for the need for colposcopy underwent colposcopy imaging and biopsy, and those who were pathologically confirmed were eligible for our study. We excluded patients who lacked definitive pathological results, and we used the WHO classification system: normal/benign, low-grade squamous intraepithelial lesion (LSIL), HSIL, and cancer. All pathology slides of punch biopsies were reviewed by pathologists from SZMCHH. Any disagreement was resolved by a panel of expert pathologists.

**Fig. 1:**
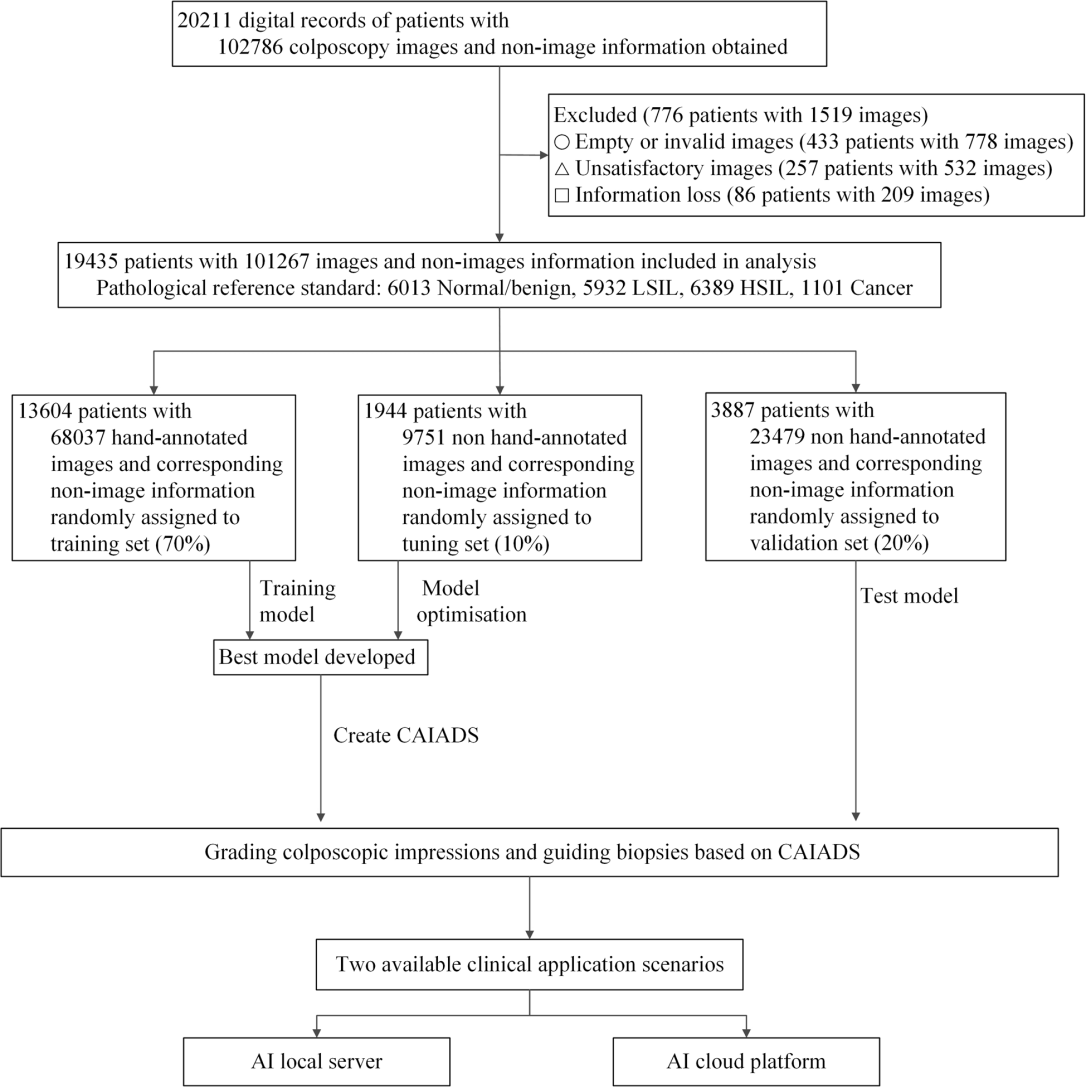
Flowchart of the development and validation of the CAIADS. Note: Circle indicates some patients’ images were excluded if colposcopists captured non-cervical invalid images. Triangle indicates for some patients’ images, the trained evaluators excluded unsatisfactory images due to poor focus, blood obscuring the cervical, vaginal wall prolapse, or other factors. Square indicates some digital records of patients were excluded due to information loss, for example lacking of colposcopy indications or/and pathological results. Non-image information included the patient’s primary screening findings (cytology, HPV status). Abbreviations: LSIL, low-grade squamous intraepithelial lesion; HSIL, high-grade squamous intraepithelial lesion; CAIADS, Colposcopic Artificial Intelligence Auxiliary Diagnostic System

The digital records of each patient were split into two categories: (1) those records containing at least five satisfactory colposcopic images commonly with ordinal timeslots (around 0 s, 60 s, 90 s, 120 s, and 150 s) and (2) those records containing non-image (cytology and HPV status), and quality control information conducted by trained evaluators, for which the exclusion criteria are shown in Fig. 1. Sample images in JPEG formats are shown in Additional file 1, Figure S1. The quality control and the complete data were randomly sampled by the severity distribution of pathological results and then assigned to a training and a tuning set for developing CAIADS and to a validation set for evaluating performance in a ratio of 7:1:2. The three datasets are obtained by random sampling according to the patient IDs, which means the patients in the validation set will not be used in the training phase.

For the training set, all selected images were automatically uploaded to an online cervix image annotation tool. These images were analyzed by a group of eight experienced colposcopists from SZMCHH. They carefully manually delineated the lesion areas and biopsy sites near the squamocolumnar junction of the cervical regions, labeling each based on the corresponding biopsy sites of the pathological results. The pathological results were the gold standard. These analyses were supervised by expert colposcopists from the National Cancer Center. The details of annotation and the annotation tool are shown in Additional file 1, Figure S2. For the tuning and validation sets, we had no manual annotations on the images. For all datasets, we made no changes to non-image information.

### Development of the CAIADS algorithm

Because colposcopists analyze both images and non-image information (cytology and HPV status) during colposcopic examinations, we developed CAIADS to simulate the diagnostic judgment of colposcopists as accurate as possible. The CAIADS algorithm consists of two deep-learning-based modules for grading colposcopic impressions and guiding biopsies, respectively. A detailed description of the CAIADS algorithm is presented in Additional file 1, Supplementary Method and Figure S3 [18–20]. Briefly, the proposed CAIADS first detected the cervical area of images for the subsequent feature extraction. Then, the extracted features were fused by a graphical convolutional network. Finally, the non-image information was concatenated to the fused features of the images to yield the result of grading impressions. Additionally, the CAIADS also predicted the suspected lesion areas to limit the range for guiding biopsy sites.

The pipeline for colposcopic grading consisted of cervix detection, feature extraction, and feature fusion networks, whereas a U-Net [21] and a YOLO [22] were implemented for lesion area segmentation and biopsy site guiding, respectively. Because an accurate lesion area segmentation can effectively reduce the number of unnecessary biopsy sites that fall outside regions containing lesions, we implemented a semi-supervised framework, as shown in Additional file 1, Figure S4. The purpose of this framework was to utilize the tuning set (only with the image-level label) to further boost the segmentation performance of CAIADS. The semi-supervised framework developed on the training set was used to produce pseudo-labels for the tuning set. Then, the tuning set with pseudo-labels was mixed with the training set to fine-tune the U-Net. A subset was separated from the training set to monitor the performance of deep-learning networks during training and to prevent overfitting. Training of the system was halted if no performance increase was observed on the separated subset.

### Validation of the CAIADS performance

We compared the agreement of colposcopic impressions of the CAIADS and original colposcopic interpretation by using pathology as the gold standard. The original colposcopic interpretation was determined and recorded by colposcopists based on the assessment of the patient’s images and non-image information. In addition, the diagnostic performance of CAIADS at different hypothetical biopsy thresholds (low-grade or worse and high-grade or worse) for the detection of pathological HSIL+ was evaluated from three aspects. Firstly, we investigated whether the diagnostic performance of CAIADS could be improved by additional non-image information, compared with grading the images alone. Secondly, we compared the performance of the CAIADS at the biopsy threshold of low-grade impression or worse versus high-grade impression or worse. Thirdly, the performance of CAIADS was compared with the original colposcopic interpretation by colposcopists. Finally, we tested the accuracy of the CAIADS in predicting biopsy sites compared with ground truth biopsy sites.

### Statistical analyses

The ROC curve was created by plotting the true positive rate (sensitivity) against the false positive rate (1–specificity), and we calculated AUC values. The diagnostic AUC value, accuracy, sensitivity, specificity, positive predictive value (PPV), and negative predictive value (NPV) were evaluated together using 95% confidence intervals (CIs) by the Clopper-Pearson method. We defined the main metric as agreement with the pathological gold standard, measured using kappa values. The McNemar test was used to evaluate the differences in diagnostic performance including agreement, accuracy, sensitivity, and specificity. A *p* value less than 0.05 (two-sided) was considered to be statistically significant. Statistical analyses were done using SAS 9.4 software (SAS Institute Inc., Cary, NC, USA), Python 3.6, and scikit-learn [23].

## Results

### Study participants

In total, 101,267 colposcopic images and non-image information from 19,435 patients were included in this study. The complete training set consisted of 68,037 images as well as non-image information from 13,604 patients with pathological results of normal/benign (*n* = 4217), LSIL (*n* = 4150), HSIL (*n* = 4489), and cancer (*n* = 748). The tuning set consisted of 9751 images as well as non-image information from 1944 patients with pathological results of normal/benign (*n* = 591), LSIL (*n* = 594), HSIL (*n* = 630), and cancer (*n* = 129). The validation set consisted of 23,479 images as well as non-image information from 3887 patients with pathological results of normal/benign (*n* = 1205), LSIL (*n* = 1188), HSIL (*n* = 1270), and cancer (*n* = 224). Detailed information of the training, tuning, and validation sets is summarized in Table 1 and Fig. 1.

**Table 1:** Basic characteristics

Characteristic	No. (%)		
	Training set	Tuning set	Validation set
Images, total no.	68,037	9751	23,479
Patients, total no.	13,604	1944	3887
Age (years)			
24–29	2694 (19.8)	399 (20.5)	988 (25.4)
30–49	8815 (64.8)	1211 (62.3)	2058 (53.0)
50–65	2095 (15.4)	334 (17.2)	841 (21.6)
Referral colposcopy indications^a^			
Primary screening results^b^	9989 (73.4)	1254 (64.5)	2337 (60.1)
Suspicious clinical symptoms^c^	3687 (27.1)	735 (37.8)	1650 (42.4)
Distribution of pathological results^d^			
Normal/benign	4217 (31.0)	591 (30.4)	1205 (31.0)
LSIL	4150 (30.5)	594 (30.6)	1188 (30.5)
HSIL	4489 (33.0)	630 (32.4)	1270 (32.7)
Cancer	748 (5.5)	129 (6.6)	224 (5.8)

*Abbreviations*: *HR-HPV* high-risk human papillomavirus, *ASC-US* atypical squamous cells of undetermined significance, *LSIL* low-grade squamous intraepithelial lesion, *HSIL* high-grade squamous intraepithelial lesion

^a^The percentages sum to over 100% due to the overlap of different colposcopy indications

^b^Primary screening results mainly included cytology (HSIL, LSIL, ASC-US, negative) and/or HPV status (HR-HPV positive without genotyping, HPV16/18, other HR-HPV positive, negative)

^c^Suspicious clinical symptoms mainly included abnormal genital tract bleeding, suspicious cervical abnormality, unexplained cervicovaginal discharge, and other factors

^d^When multiple lesions were present in a patient, the highest grade was used as the final pathological diagnosis

### The colposcopic grading performance

**Fig. 2:**
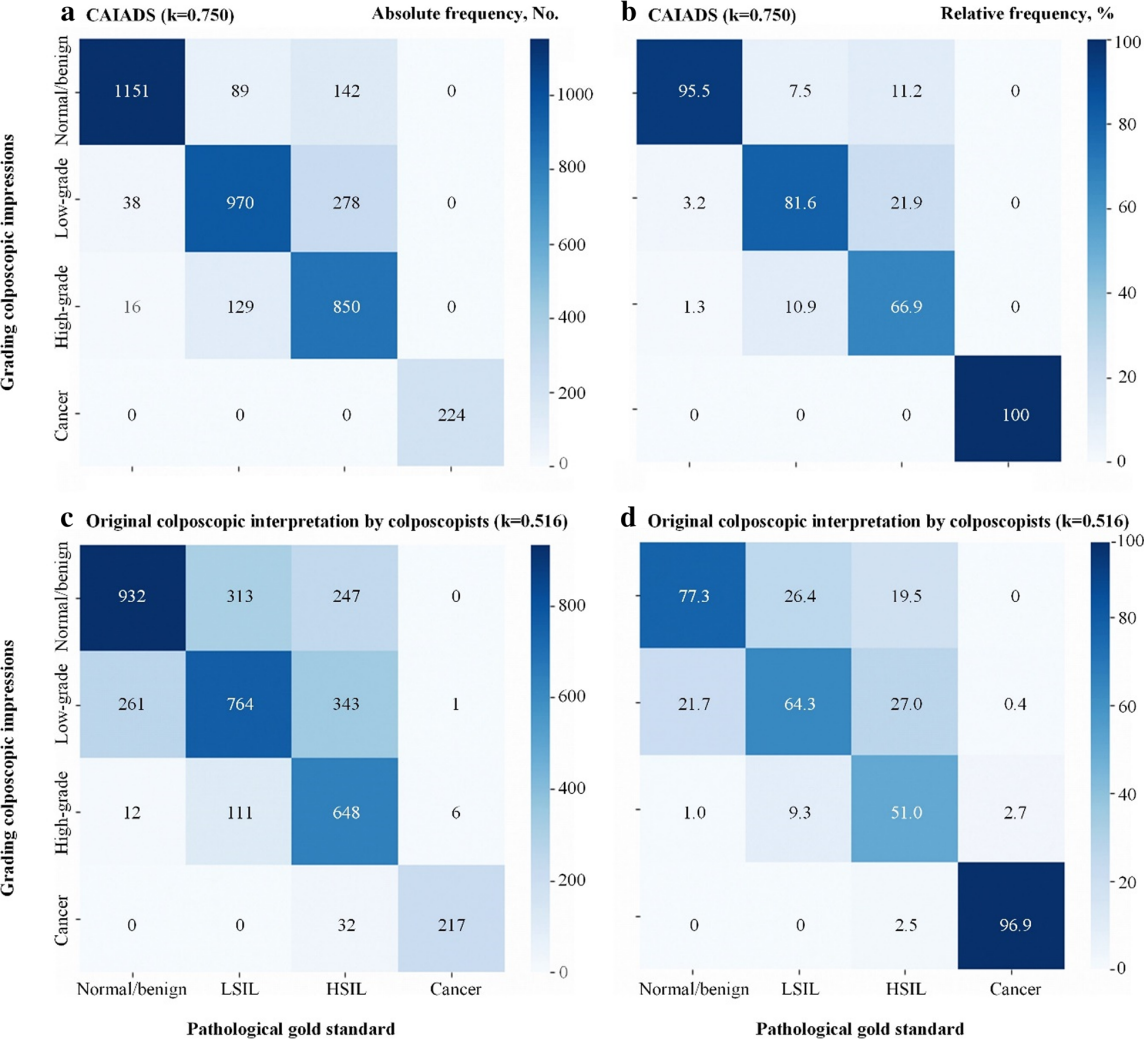
Confusion matrices of colposcopic grading distribution. Note: Data are shown for the validation set. The agreement of CAIADS (**a**, **b**) graded colposcopic impressions and original colposcopic interpretation by colposcopists (**c**, **d**) with the pathological gold standard is shown in the kappa value above each matrix. Absolute frequency (**a**, **c**) and relative frequency (**b**, **d**) are shown. For the relative frequency, the number of cases in each unit is divided by the total cases in each column. Abbreviations: LSIL, low-grade squamous intraepithelial lesion; HSIL, high-grade squamous intraepithelial lesion; CAIADS, Colposcopic Artificial Intelligence Auxiliary Diagnostic System

Of the 3887 patients in the validation set, the CAIADS achieved an overall agreement of 82.2% for grading colposcopic impressions with the pathological gold standard (kappa 0.750). For patients pathologically confirmed as normal/benign, LSIL, HSIL, and cancer, the agreements between colposcopic impressions of the CAIADS and pathology were 95.5%, 81.6%, 66.9%, and 100%, respectively. A review of discrepant cases revealed that disagreement most often occurred when grading LSIL (81.6%) and HSIL (66.9%). Moreover, we observed that the overall agreement of the CAIADS-graded colposcopic impressions and pathology was higher than that of the original colposcopy interpretation by the colposcopists (82.2% versus 65.9%, kappa 0.750 versus 0.516, *p* < 0.001). Confusion matrices of colposcopic grading distribution are presented in Fig. 2.

### Pathologically HSIL+ diagnostic performance

**Fig. 3:**
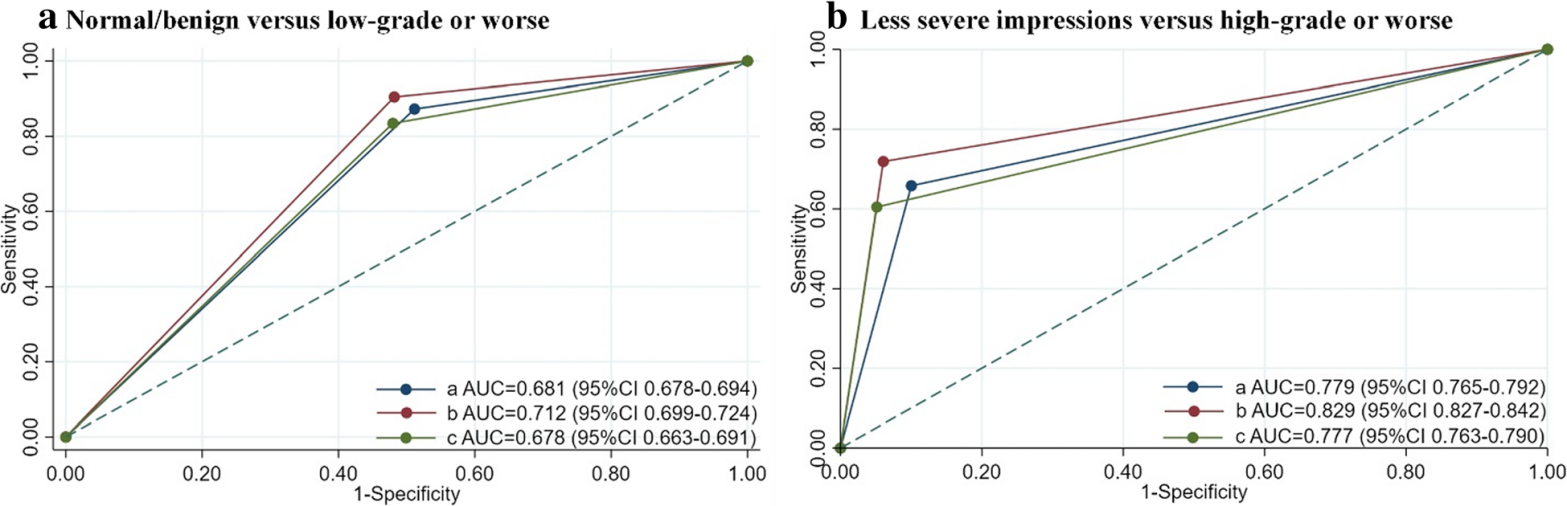
The ROC curve of diagnostic performance for detecting pathological HSIL+ at different hypothetical biopsy thresholds. Note: The hypothetical biopsy thresholds are shown: **A) **normal/benign versus low-grade or worse and **B)** less severe impressions versus high-grade or worse. **a** Analysis of images alone by CAIADS. **b** Analysis of both images and non-image information by CAIADS. **c** Analysis of both images and non-image information by colposcopists. Abbreviations: CAIADS, Colposcopic Artificial Intelligence Auxiliary Diagnostic System; AUC, area under the curve. Less severe impressions included normal/benign and low-grade. Non-image information included the patient’s primary screening findings (cytology, HPV status)

Of the 3887 patients in the validation set, there were 1494 pathologically confirmed HSIL+. The diagnostic performance of the CAIADS at different biopsy thresholds for detecting HSIL+ was evaluated from three aspects, as shown in Table 2 and Fig. 3. Firstly, compared with grading the images alone, the analyses of additional non-image information improved the diagnostic performance of CAIADS. The area under the curve (AUC) values were statistically significantly increased at different biopsy thresholds (low-grade or worse 0.681, 95% CI 0.678–0.694 versus 0.712, 0.699–0.724; high-grade or worse 0.779, 0.765–0.792 versus 0.829, 0.827–0.842; all *p* < 0.001), the accuracies (low-grade or worse 63.6%; 95% CI 62.1–65.1% versus 66.7%, 65.2–68.2%; high-grade or worse 80.7%, 79.4–81.9% versus 85.5%, 84.3–86.6%; all *p* < 0.05), the sensitivities (low-grade or worse 87.3%, 95% CI 85.5–88.9% versus 90.5%, 88.9–91.4%; high-grade or worse 65.8%, 63.3–68.2% versus 71.9%, 69.5–74.2%; all *p* < 0.001), and the specificities (low-grade or worse 48.9%, 95% CI 46.8–50.9% versus 51.8%, 49.8–53.8%, *p* = .04; high-grade or worse 90.0%, 88.7–91.2% versus 93.9%, 92.9–94.9%, *p* < 0.001). Secondly, the rating of the biopsy threshold of low-grade or worse was statistically more sensitive (90.5%, 95% CI 88.9–91.4% versus 71.9%, 69.5–74.2%, *p* < 0.001), albeit less specific than those rated high-grade or worse (51.8%, 95% CI 49.8–53.8% versus 93.9%, 92.9–94.9%, *p* < 0.001). Thirdly, we observed that the AUC values of the CAIADS were higher than the original colposcopy interpreted by colposcopists using either a biopsy threshold (low-grade or worse 0.712, 0.699–0.724 versus 0.678, 0.663–0.691; high-grade or worse 0.829, 0.827–0.842 versus 0.777, 0.763–0.790; all *p* < 0.001), the accuracies (low-grade or worse 66.7%, 95% CI 65.2–68.2% versus 64.1%, 62.6–65.6%; high-grade or worse 85.5%, 84.3–86.6% versus 81.6%, 80.4–82.8%; all *p* < 0.05), and the sensitivities (low-grade or worse 90.5%, 95% CI 88.9–91.4% versus 83.5%, 81.5–85.3%; high-grade or worse 71.9%, 69.5–74.2% versus 60.4%, 57.9–62.9%; all *p* < 0.001), whereas the specificities were similar (low-grade or worse 51.8%, 95% CI 49.8–53.8% versus 52.0%, 50.0–54.1%, *p* = 0.91; high-grade or worse 93.9%, 92.9–94.9% versus 94.9%, 93.9–95.7%, *p* = 0.17). Overall, the CAIADS achieved higher diagnostic sensitivity and similar specificity compared with the original colposcopy interpreted by colposcopists for detecting HSIL+.

**Table 2:** The diagnostic performance for detecting pathological HSIL+ at different hypothetical biopsy thresholds

	Accuracy, % (95% CI)	Sensitivity, % (95% CI)	Specificity, % (95% CI)	Positive predictive, value % (95% CI)	Negative predictive, value % (95% CI)
**Analysis of images alone by CAIADS**					
Normal/benign versus low-grade or worse	63.6 (62.1–65.1)	87.3 (85.5–88.9)	48.9 (46.8–50.9)	51.6 (49.6–53.6)	86.0 (84.1–87.8)
Less severe impressions^a^ versus high-grade or worse	80.7 (79.4–81.9)	65.8 (63.3–68.2)	90.0 (88.7–91.2)	80.4 (78.0–82.6)	80.8 (79.3–82.3)
**Analysis of both images and non-image information**^**b**^ **by CAIADS**					
Normal/benign versus low-grade or worse	66.7 (65.2–68.2)	90.5 (88.9–91.4)	51.8 (49.8–53.8)	54.0 (52.0–55.9)	89.7 (88.0–91.3)
Less severe impressions^a^ versus high-grade or worse	85.5 (84.3–86.6)	71.9 (69.5–74.2)	93.9 (92.9–94.9)	88.1 (86.2–89.9)	84.3 (82.8–85.6)
**Analysis of both images and non-image information**^**b**^ **by colposcopists**					
Normal/benign versus low-grade or worse	64.1 (62.6–65.6)	83.5 (81.5–85.3)	52.0 (50.0–54.1)	52.1 (50.0–54.1)	83.5 (81.5–85.3)
Less severe impressions^a^ versus high-grade or worse	81.6 (80.4–82.8)	60.4 (57.9–62.9)	94.9 (93.9–95.7)	88.0 (85.9–89.9)	79.3 (77.8–80.8)

*Abbreviations*: *CAIADS* Colposcopic Artificial Intelligence Auxiliary Diagnostic System

^a^Less severe impressions included normal/benign and low-grade

^b^Non-image information included patient’s primary screening findings (cytology, HPV status)

### Biopsy sites predicting performance

**Fig. 4:**
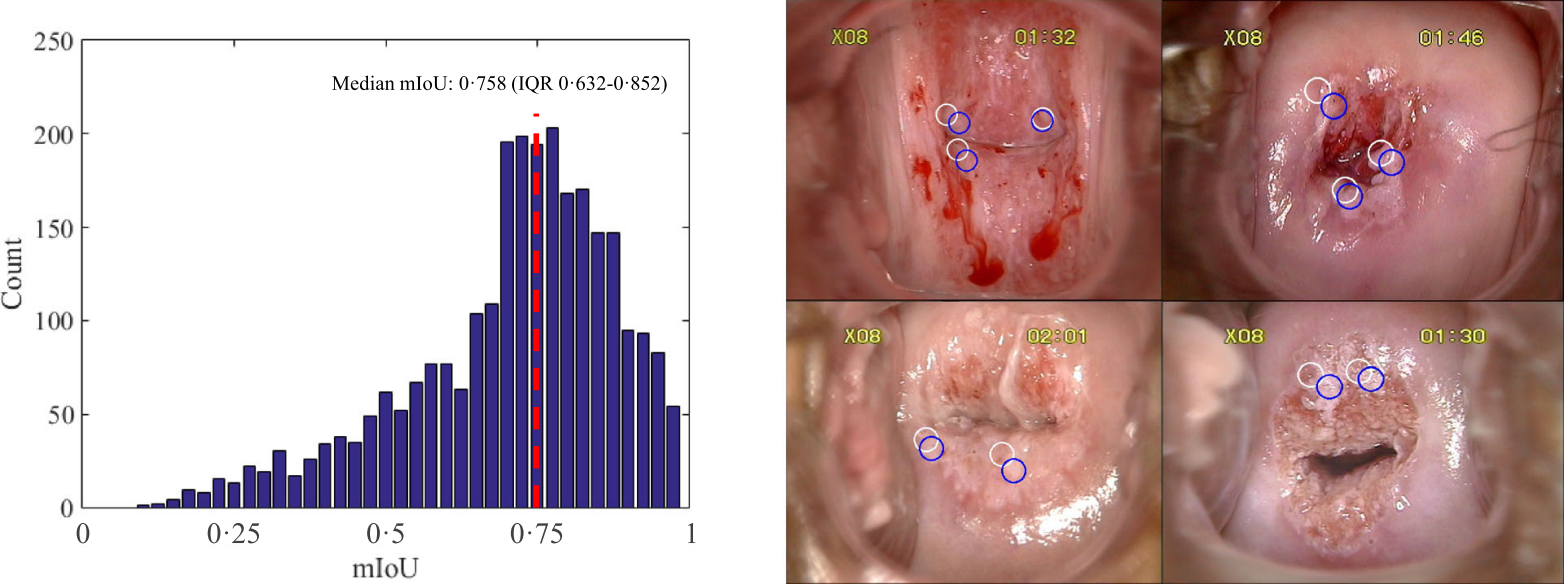
The accuracy of the CAIADS in predicting biopsy sites. Note: We tuned off the displays of internal structure effects to allow more straightforward comparison between CAIADS and ground truth biopsy sites. Left, the distribution of mean-intersection-over-union (mIoU) for the validation set. Right, various examples of CAIADS for predicting biopsy sites. The white and blue circle-shaped sites represented the predicted and ground truth biopsy sites, respectively. Abbreviations: CAIADS, Colposcopic Artificial Intelligence Auxiliary Diagnostic System; IQR, interquartile range

The accuracy of the CAIADS in predicting biopsy sites was evaluated using ground truth biopsy sites. Figure 4 shows the distribution of mean-intersection-over-union (mIoU) for the validation set, and various examples of biopsy sites predicted by CAIADS. A median mIoU of 0.758 (interquartile range 0.632–0.852) was achieved by CAIADS on the validation set. The higher mIoU value represented the more accurate performance for biopsy site prediction. The white and blue circle-shaped sites represent the predicted and ground truth biopsy sites, respectively.

## Discussion

The performance of colposcopy and directed biopsies is the major challenge in the cervical cancer screening process. Previous studies [24, 25] have suggested that even some experienced colposcopists are challenged at correctly grading colposcopic impressions. To this end, we developed a Colposcopic Artificial Intelligence Auxiliary Diagnosis System which we termed CAIADS. This system was trained and validated in 101,267 retrospective colposcopic images as well as in non-image information from 19,435 patients.

To the best of our knowledge, this is the first study using a large-scale dataset in the field of artificial intelligence-graded colposcopic impressions and guided biopsies worldwide. The CAIADS method achieved a high level of agreement (82.2%) with pathological results as the gold standard regarding grading all colposcopic impressions and was higher than the original colposcopic interpretation by colposcopists (65.9%). This level of agreement was significantly higher than that of Benedet et al. (52.0%) [26] which used a large dataset of colposcopic diagnostic studies from 84,244 British patients. In addition, we observed that CAIADS had an excellent agreement with pathology results when distinguishing between normal/benign (95.5%) and cancer (100%), thereby providing more reassurance in regard to the veracity of positive results and negative results. Despite the fact that AI was not limited by diagnostic subjectivity of colposcopists, as expected, CAIADS showed promising but suboptimal performance in grading LSIL (81.6%) and HSIL (66.9%) owing to the vague and subtle distinctions between LSIL and HSIL features (the thickness of acetowhite epithelium, vascular patterns, margins/border, etc.). For example, some LSIL lesions are condylomas caused by HPV infection, but only in a very small range contains HSIL, which it is difficult to judge. The ability to distinguish between LSIL and HSIL remains one of the most important challenges in colposcopy practice as well as for colposcopists. However, in terms of AI characteristics in iterative enhancement capability, continual receiving and learning would increase the discrimination power of the CAIADS between LSIL and HSIL. Therefore, it should be persistently and closely observed and evaluated in further research.

In real-world clinical practice, the most important task for colposcopists is to guide colposcopic biopsy for detecting underlying HSIL+ cases for subsequent treatment. Biopsy protocols remain varied for colposcopists, and the option of biopsy thresholds remained controversial [5, 27]. In our study, the diagnostic performance of the CAIADS at different hypothetical biopsy thresholds for the detection of pathological HSIL+ was evaluated from three aspects. Firstly, the statistically significant improvements for HSIL+ detection suggested that the diagnostic performance of CAIADS can be improved by additional non-image information. As such, CAIADS could have the potential to perform a comprehensive evaluation by analysis of both images as well as non-image information for detecting HSIL+. This could reduce the risk of misdiagnosis and provide tailored colposcopic examinations individually, based on the principle of precision prevention. Secondly, we found that the identification of low-grade or worse lesions was a highly sensitive indicator for detecting HSIL+, compared with the biopsy threshold at high-grade or worse. The colposcopy-guided biopsies were required to guide subsequent treatment or management. Although the specificity would be unsatisfactory as 51.8% that some of the patients with low-grade colposcopic impression may not have HSIL+, minimizing false negative values for HSIL+ should be a priority in choosing an appropriate cutoff point. In addition, cost-effectiveness should be considered, given the high cost of cervical cancer treatment. Thirdly, the CAIADS achieved higher sensitivity and similar specificity compared with the original colposcopic interpretation by the colposcopists in detecting HSIL+. These findings suggest that CAIADS has potential applications in assisting beginners with diagnoses, because the system extracted and learned mass and robust cervical lesion features from annotated images in terms of the pathological reports. Given the important role of choosing whether and where to place cervical biopsy sites to detect underlying disease states, automatic biopsy localization is of clinical importance. In the validation set, the CAIADS achieved a median mIoU of 0.758, which demonstrated the CAIADS could be implemented as an auxiliary biopsy location tool for colposcopists.

On the basis of robustness of CAIADS in grading colposcopic impressions and guiding biopsies, we propose the integration of CAIADS into local colposcopy clinics as an accurate and auxiliary diagnosis tool for colposcopists during colposcopic procedures. We also propose establishing a cloud-based artificial intelligence platform to provide accessible telemedical assistance for most low-resource settings, such as China (accounting for 20% of the world’s population), where experienced colposcopists and colposcopy services are in short supply. Therefore, CAIADS may be expected to fill a need for standardized cervical cancer screening/diagnosis procedures, narrow the gap of diagnostic ability between tertiary hospitals and primary care hospitals, improve the quality of screening programs, and promote cooperation in scaling up coverage worldwide. Currently, CAIADS is being routinely introduced into the colposcopic clinical workflow with real-time assistance at SZMCHH, and it has been recently implemented by other hospitals, providing free access to the cloud-based artificial intelligence-aided colposcopic examination.

There are several limitations. First, although CAIADS showed satisfactory accuracy in the validation set, the design was retrospective. In this regard, a prospective study will be conducted to further validate its performance and provide evidence of cost-effectiveness in the clinical practice of cervical cancer screening. Second, we focused on the grading of colposcopic impressions for cervical neoplasia lesions in the current study. However, miscellaneous findings such as polyps, stenosis, and condyloma should be identified and biopsied in some cases. Therefore, CAIADS cannot replace clinician evaluations in grading colposcopic impressions and guiding biopsies but could assist colposcopists in clinical practice. CAIADS could be particularly helpful for less experienced colposcopists who practice in LMICs. In future studies, we will be considering the recruitment of patients with miscellaneous findings such as polyps, stenosis, and condyloma, to extract their lesion features for further training and validation of the CAIADS algorithm.

## Conclusions

The CAIADS achieved higher sensitivity and comparable specificity to colposcopies interpreted by colposcopists and also demonstrated satisfactory accuracy in guiding biopsy sites. The CAIADS has potential in assisting beginners and for improving the diagnostic quality of colposcopy and biopsy in the detection of cervical precancer/cancer.

## Supplementary Information

**Additional file 1: Table S1.** Participating study sites. **Table S2.** Detailed information of E-GCN architecture. **Table S3.** Look-up matrix mapping non-image information to features. **Figure S1.** Examples of colposcopy images for increasingly severe pathology. **Figure S2.**. Cervix image annotation tool. **Figure S3.** Pipeline of system for colposcopic grading and guiding biopsies. **Figure S4.** Pipeline of semi-supervised learning framework for lesion segmentation.

## Data Availability

The datasets used and/or analyzed during the current study are available from the corresponding author on reasonable request.

## Abbreviations

AI: Artificial intelligence
ASCCP: American Society for Colposcopy & Cervical Pathology
AUC: Area under the curve
CAIADS: Colposcopic Artificial Intelligence Auxiliary Diagnostic System
CIs: Confidence intervals
HPV: Human papillomavirus
HSIL+: High-grade squamous intraepithelial lesion or worse
IRB: Institutional review board
LMICs: Low- and middle-income countries
LSIL: Low-grade squamous intraepithelial lesion
mIoU: Mean-intersection-over-union
NPV: Negative predictive value
PPV: Positive predictive value
SZMCHH: Shenzhen Maternity & Child Healthcare Hospital
WHO: World Health Organization
