# Supplementary material for: Development and validation of an artificial intelligence system for grading colposcopic impressions and guiding biopsies
Source: BMC Med. 2020 Dec 22;18:406. doi: 10.1186/s12916-020-01860-y (PMC7754595; doi:10.1186/s12916-020-01860-y)
Supplement: Supplementary file 1 — Additional file 1: Table S1. Participating study sites. Table S2. Detailed information of E-GCN architecture. Table S3. Look-up matrix mapping non-image information to features. Figure S1. Examples of colposcopy images for increasingly severe pathology. Figure S2.. Cervix image annotation tool. Figure S3. Pipeline of system for colposcopic grading and guiding biopsies. Figure S4. Pipeline of semi-supervised learning framework for lesion segmentation. [file 12916_2020_1860_MOESM1_ESM.docx]

Supplementary online for

**Development and validation of an artificial intelligence system for grading colposcopic impressions and** **guiding biopsies**

**This supplementary material includes:**

Supplementary Methods

Table S1: Participating study sites.

Table S2: Detailed information of E-GCN architecture.

Table S3: Look-up matrix mapping non-image information to features.

Figure S1: Examples of colposcopy images for increasingly severe pathology.

Figure S2: Cervix image annotation tool.

Figure S3: Pipeline of system for colposcopic grading and guiding biopsies.

Figure S4: Pipeline of semi-supervised learning framework for lesion segmentation.

**Supplemental Methods**

**Development of the CAIADS algorithm**

The CAIADS algorithm consisted of several deep learning networks for different purposes, which were cervix detection, feature encoding, graph convolutional network (GCN) based feature fusion, and lesion area segmentation networks. The CAIADS algorithm mapped input features (colposcopic images and non-image information) to the corresponding two target tasks (i.e., grading colposcopic impressions and guiding biopsies). The model iteratively learned hierarchical representations from the input image and non-image information. As shown in Figure S3, the non-image information involved in this study was primary screening findings (cytology and HPV status). The originality of our proposal can be concluded into three folds: First, compared to the previous studies using single colposcopic image for automated cervical cancer diagnosis, our framework uses a graph convolutional network to describe the relationship between cervical images captured on different time slots, which imitates the clinical judgement of colposcopist and achieves more accurate classification performance; Second, non-image information (cytology and HPV status) is embedded and fused with the features extracted from cervical images for an end-to-end training. To our best knowledge, this is the first work boosting the classification accuracy of AI approach by embedding rich clinical information; Last but not least, our framework consists of a complete pipeline for automated diagnosis of cervical cancer, including colposcopic impression grading, lesion area segmentation and biopsy site guiding, which comprehensively aids colposcopists for the more accurate clinical judgement.

**a. Cervix detection**

As the cervix may shift during the image acquisition, a convolutional neural network (CNN) was trained to crop cervix region from colposcopic images. For training and validation of cervix detection network, the cervix location of each image was manually annotated by drawing a rectangular bounding box around the cervix. The cervix detection task, which had only one cervix bounding box, was much easier than the common object detection task with multiple targets. Therefore, we applied a simpler approach to address this problem, instead of exploiting the complicated existing methods such as Mask R-CNN and YOLO. The widely-used CNN-ResNet-50-was employed as the backbone to directly predict the cervix bounding box. The fully connected (FC) layer of ResNet-50-originally with 1000 neurons-was replaced with a new FC layer with four neurons, which corresponded to the coordinate of the left-top corner, and the width and height (x, y, w, h) of the cervix bounding box. The cervix detection network was supervised by the L_1_ loss. Let (a, b, p, q) denoted the ground truth of the cervix bounding box, the L_1_ loss for the regression of coordinate and box size can be written as:

$L_{1}=||x-a||+||y-b||+||w-p||+||h-q||$.

**b. Feature encoding network**

We separately trained five feature encoding networks to fully extract features from the cervix regions of five images with ordinal timeslots (around 0s, 60s, 90s, 120s, and 150s). The ResNet-101 was adopted as the backbone of the feature encoding network. For a better feature extraction, the feature encoding networks were initialized using ImageNet pre-trained weights and then separately finetuned with corresponding time-slot images under the supervision of cross-entropy loss. After all the feature encoding networks were well-trained, the outputs of the second-to-last layer were employed as the encoded features – 2048 × 1 × 1 vector. The encoded features contained the representative information of the whole sequential images, which would be sent to the following GCN based fusion network. To demonstrate diagnostic effectiveness of using five time-slot images, we tested each of the feature encoding networks on the validation set. The CAIADS was observed to surpass the best single model, i.e., trained with 120s images, by a large margin (around 5% for mean classification accuracy).

**c. Feature fusion network**

The features extracted from sequential images were sent to the following GCN based feature fusion component. A brief introduction of GCN can be found in a recent survey. The GCN set the encoded features as the node features and used learnable edge features to represent the relationships between nodes, which provided a more flexible graph topology. The detailed information of E-GCN was presented in Table S2. As our GCN was a directed graph, the edge features ($X^{e}$) had the property: $X_{ij}^{e} \left( i\to j \right)\neq X_{ji}^{e} \left( j\to i \right)$. Therefore, there were 20 edges in the constructed graph. A 1 × 1 learnable weight was assigned to each edge, which led $X^{e}$ to have a shape of 1 × 1 × 20. The shape of encoded feature was 1 × 1 × 2048 for each graph node. Hence, the input sizes of GCN were $X_{i}^{v} \to1\times1\times2048$ and $X^{e} \to1\times1\times20$, respectively. The GCN involved four layers, and the $f_{n}^{1}$ was mainly used to compact the node features, while the rest three layers ($f_{n}^{2}$, $f_{e}^{1}$, and $f_{e}^{2}$) were responsible for features fusion and graph topology tuning. The updated graph was finally flattened to a 640-d (128 × 5) feature vector containing images information. For a more accurate cervical grading prediction, the non-image information (patient’s primary screening findings) was embedded and concatenated to the features of images for a joint training. Specifically, the non-image information was embedded into a feature space using a look-up map as presented in Table S3, which resulted in three 4-d one-hot feature vectors. The primary screening findings included cytology and HPV status. Then, the four feature vectors were concatenated to the flattened image features, leading to a 656-d feature, and connected to a fully-connected layer, which produced a 1 × 1 vector for automated grading of colposcopic impressions (normal/benign, low-grade, high-grade, and cancer). The training process was supervised by the cross-entropy loss (*L_cross-entropy_*), which was defined as:

$$L_{cross-entropy}=\frac{1}{N}\sum_{i} L_{i}=\frac{1}{N}\sum_{i} -log(\frac{e^{f_{y_{i}}}}{\sum_{j} e^{f_{j}}})$$

where $f_{j}$ denoted the *j*-th element ($j\in[1,K]$, *K=4* is the number of classes) of vector of class scores *f*, $y_{i}$ was the label of *i*-th input feature and *N* is the number of training data.

**d. Lesion area segmentation network**

A fully convolutional network-U-Net-was adopted in the CAIADS to perform lesion area segmentation. The U-Net had an encoder-decoder architecture. The encoder mainly extracted the visual feature representation from the cervical images, while the decoder was responsible to disentangle the encoded features and generate the pixel-wise segmentation result. Some short-cut connections were established between the encoder and decoder for flexible flow of information. The images whose lesion areas and biopsy sites had been manually annotated, were used to train the lesion segmentation U-Net and biopsy site guiding YOLO, respectively. Within the lesion areas, YOLO was used to localize the biopsy sites. As YOLO (YOLOv3-416 was used in our system) yielded the bounding boxes for object detection, we used the bounding boxes of circles centered on the biopsy sites as the supervision signals. The predicted lesion areas were used to limit the range for biopsy site localization.

**Data augmentation**

To augment the training set, several transformations were adopted, which included horizontally or vertically flipping the images and rotating the images by 90 degrees. These transformations were performed at random during the training process-three probabilities were randomly generated for each image and the corresponding transformation was performed when the possibility was large than 50%.

**Training details**

The three components of the proposed AI model (cervix detection, feature encoding and feature fusion) were trained with a consecutive manner-the prior network was trained and fixed for the training of the latter one. The lesion area segmentation network was separately trained. The cervix detection, feature encoding and lesion area segmentation networks were implemented using PyTorch, and the GCN was developed using Deep Graph Library (DGL). The four networks were trained with a mini-batch size of 16 on one GPU (Tesla P40 with 24 GB memory). The initial learning rate was set to 0.001 and the Adam solver was adopted for the optimization.

**Semi-supervised learning using tuning set**

The lesion areas predicted by the CAIADS were used to restrict the range for the automated localization of biopsy sites, i.e., the estimated biopsy sites must be located in the lesion areas. In this regard, an accurate segmentation of lesion area can effectively reduce the number of unnecessary biopsy sites, which fall outside the areas containing lesions. In this study, the data collected from six multi-centers can be separated to the training set with manual annotations of lesion area and biopsy sites, and the tuning set only with the image-level annotations, i.e., pathological results. To this end, we implemented a semi-supervised learning framework, as shown in Figure S2, to utilize the tuning data to further improve the accuracy of cervical lesion area segmentation. In particular, the model trained with training data was applied to generate the pseudo-label for the tuning data with only image-level labels. In other words, the generation of pseudo-labels is an inference process of the trained segmentation network on unlabeled data. Using the pseudo-labels, the tuning data can be included to finetune the model with annotated training data for a more accurate segmentation performance of cervical lesion areas.

**Multi-center domain adaptation**

The domain adaptation between multi-centers was one of the main challenges in this work. The images from six centers often had different imaging conditions such as color and illumination, which may degrade the generalization of the computer aided diagnosis system. To address the problem, we exploited CycleGAN for the imaging condition alignment between the multi-centers. CycleGAN had two paired generator-discriminator modules, which were capable of learning two mappings, i.e., from domain A to domain B (G_AB_, D_B_) and vice versa (G_BA_, D_A_). The generators (G_AB_, G_AB_) translated images between the source and target domains, while the discriminators (D_A_, D_B_) aimed to distinguish the real and translated data. Thereby, the generators and discriminators were gradually updated during this adversarial competition. In this study, the domains A and B were the anchor center (SZMCHH) and the rest ones, respectively. The images from multi-centers were translated to the anchor domain at the very beginning of model implementation, which significantly improved the generalization of the CAIADS. It was worthwhile to mention that the translated images were mixed with the images from the anchor center, which were then randomly separated to training, tuning and validation sets. We conducted an ablation study to evaluate the contribution made by CycleGAN domain adaptation. The experimental results showed that the mean classification accuracy of CAIADS significantly degraded without CycleGAN.

**The image annotation principles for cervical lesion areas and biopsy sites.**

Colposcopists mainly focused on the cervical regions near the squamocolumnar junction (SCJ) at the transformation zone to carefully hand-delineate appealing lesion areas and biopsy sites, assigning each with labelling based on corresponding biopsy sites of pathological results as the gold standard. When multiple lesions and biopsies were present, the highest-grade was used as the final pathological diagnosis. For annotation of cervical lesion areas, six points were summarized as follows, 1) The thickest or densest sites of acetowhite epithelium (coarse and uneven surface, gyrus-like changes), 2) The areas with the characteristics of vascular changes (punctuation vessels, mosaic vessels, atypical vessels), 3) The areas with complex features (white inlaid dichroism, white inlaid triad, white ring of glands and patches of glands), 4) The raised cervical lesion areas (nodular, cauliflower-like, polypoid or papillary pattern), 5) Internal bleeding of cervical lesion areas (ulceration and necrosis), 6) The areas of acetowhite epithelium that is “red” presented in original/pre-acetic-acid image (abundant vascularity). To label biopsy sites, colposcopists in clinical practice usually need to magnify the electronic colposcopic images from six to eight times to observe cervical changes, and using punch biopsy forceps to remove an approximately circular tissue sample with a diameter of 5mm, Therefore, based on the fact that the image has been magnified by 6-8 times, we designed a circular biopsy range with a diameter of 1cm for colposcopists to manually label the biopsy sites appearing on the cervical image, as shown in Figure S2.

**Table S1: Participating study sites.**

**Institution:**

1. Shenzhen Maternity & Child Healthcare Hospital

2. Jiangxi Maternity and Child Health Hospital

3. Chongqing University Cancer Hospital

4. Chengdu Women’s and Children’s Central Hospital

5. Liaoning Cancer Hospital & Institute

6. Affiliated Cancer Hospital of Zhengzhou University/Henan Cancer Hospital

**Table S2: Detailed information of E-GCN architecture.**

| **Input size:** $X_{i}^{v} \to1\times1\times2048$ and $X^{e} \to1\times1\times20$ | | |
| --- | --- | --- |
|  | **Architecture** | **Output size** |
| $\boldsymbol{f}_{\boldsymbol{v}}^{\boldsymbol{1}}$ | C (1, 2048, 128), ReLU | $h_{i}^{(1)}\to1\times1\times128$ |
| $\boldsymbol{f}_{\boldsymbol{e}}^{\boldsymbol{1}}$ | C (1, 20, 20), Sigmoid | $e^{(1)}\to1\times1\times20$ |
| $\boldsymbol{f}_{\boldsymbol{n}}^{\boldsymbol{2}}$ | C (1, 128, 128), ReLU | $h_{i}^{(2)}\to1\times1\times128$ |
| $\boldsymbol{f}_{\boldsymbol{e}}^{\boldsymbol{2}}$ | C (1, 20, 20), Sigmoid | $e^{(2)}\to1\times1\times1$ |

f is the GCN layer, C (k, n) represents the 1D convolutional layer with kernel size (k) and channel (n). h and e are the output node and edge features, respectively.

**Table S3: Look-up matrix mapping non-image information to features.**

|  | **^a^HPV 16/18** | **13/14 HR-HPVs** | **12 other HR-HPV** | **Negative** |
| --- | --- | --- | --- | --- |
| **One-hot feature** | 1000 | 0100 | 0010 | 0001 |
|  | **^b^HSIL+** | **^c^LSIL** | **^d^ASC-US** | **Negative** |
| **One-hot feature** | 1000 | 0100 | 0010 | 0001 |

Abbreviations: ^a^HPV, human papillomavirus; ^b^HSIL+, cytological high-grade squamous intraepithelial lesion or worse; ^c^LSIL, cytological low-grade squamous intraepithelial lesion; ^d^ASC-US, cytological atypical squamous cells of undetermined significance.

**
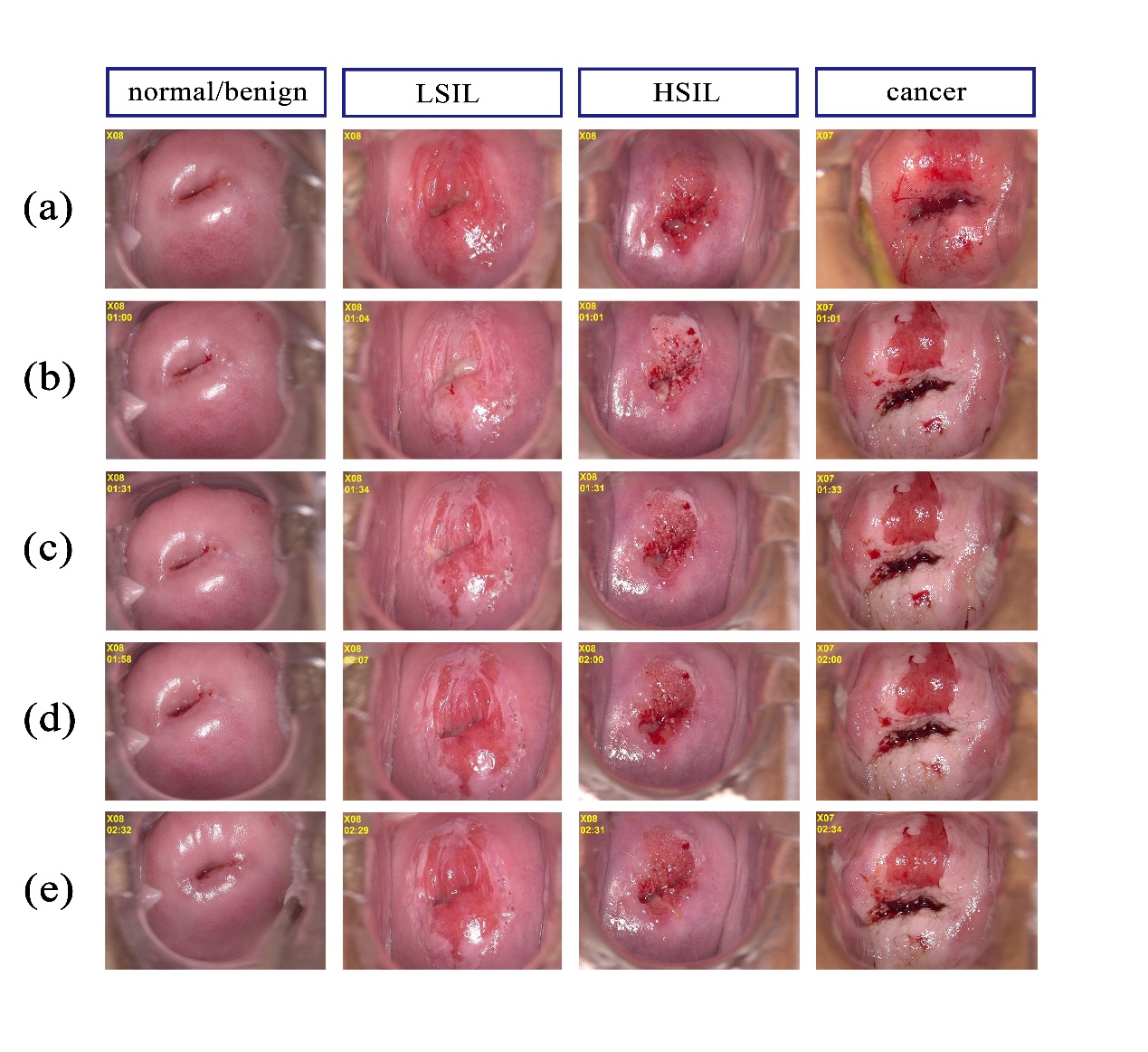
**

**Figure S1：Examples of colposcopy images for increasingly severe pathology.**

Note: These images contained examples of pathological normal/benign, low-grade squamous intraepithelial lesion (LSIL), high-grade squamous intraepithelial lesion (HSIL), and cancer. Each patient included at least five images with ordinal timeslots, (a) one original/pre-acetic-acid image at the time of 0s; (b)-(e) other four post-acetic-acid images at the time around 60s, 90s, 120s, and 150s, respectively.


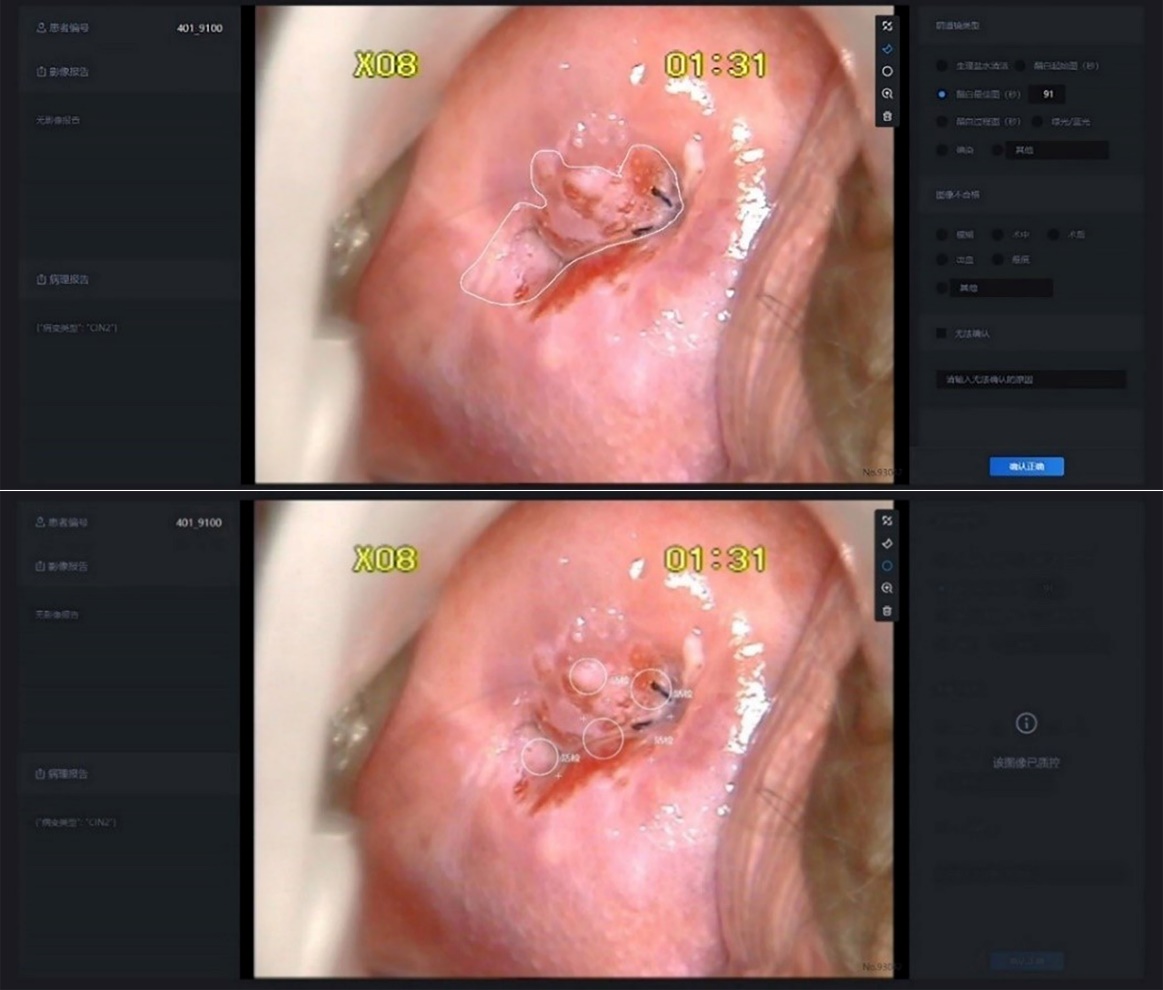


**Figure S2: Cervix image annotation tool.**

Note: The white delineation contour lines and circles were respectively used to annotate the boundaries of cervical lesion areas and biopsy sites by colposcopists.


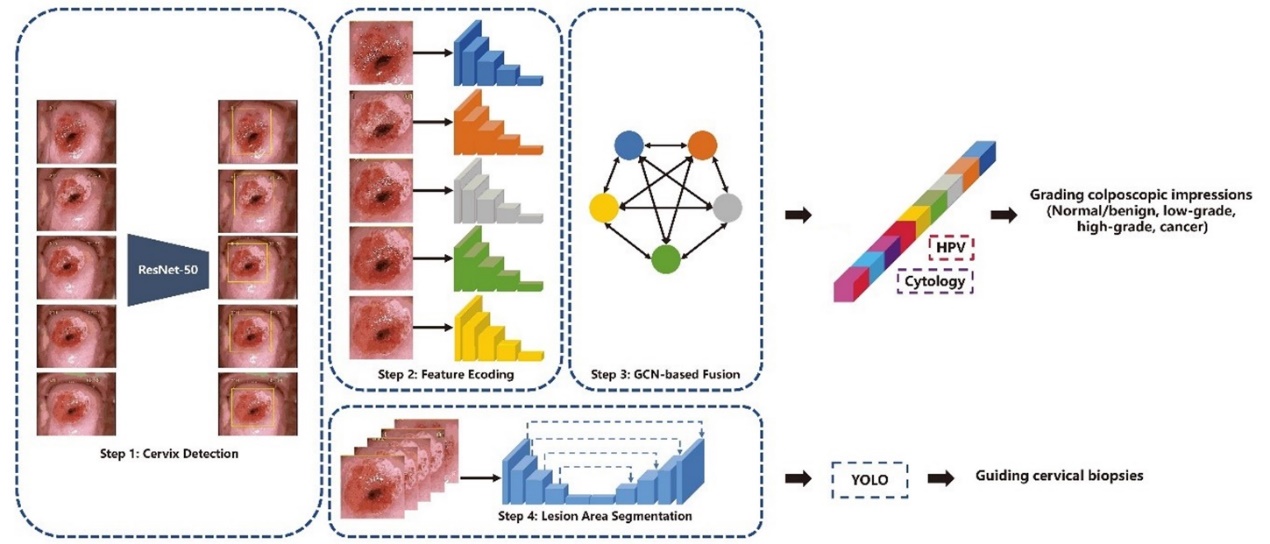


**Figure S3: Pipeline of system for colposcopic grading and guiding biopsies.**

Note: The CAIADS algorithm consisted of four deep learning networks for different purposes, including cervix detection, feature encoding, graph convolutional network (GCN) based feature fusion, and lesion area segmentation networks. The CAIADS algorithm mapped input features (images and non-image information) to the corresponding two target tasks (i.e., grading colposcopic impressions and guiding biopsies). The non-image information included primary screening findings (cytology, HPV status).


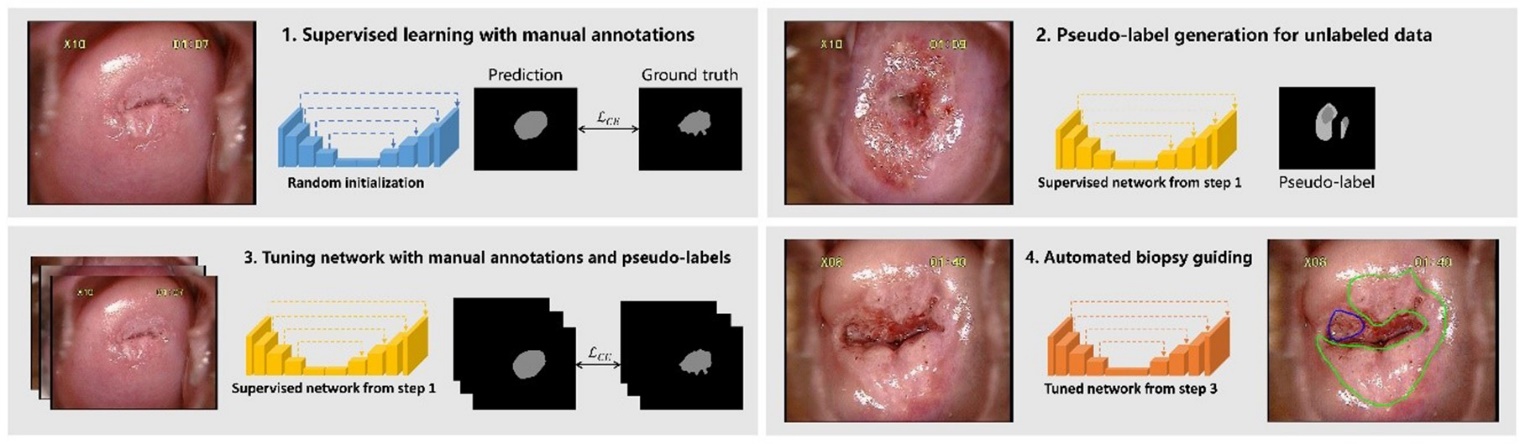


**Figure S4: Pipeline of semi-supervised learning framework for lesion segmentation.**

Note: The framework involved three steps to achieve the benefit from unlabeled data for lesion area segmentation. First, we trained a U-Net with annotated colposcopic images, and then used it to generate the pseudo-labels for the unlabeled data. Therefore, the unlabeled data can be used to finetune the U-Net together with the annotated data to further boost the segmentation accuracy of cervical lesion, which can effectively reduce the number of unnecessary predicted biopsy sites.
